# A qualitative process evaluation of a diabetes navigation program embedded in an endocrine specialty center in rural Appalachian Ohio

**DOI:** 10.1186/s12902-018-0278-7

**Published:** 2018-07-27

**Authors:** Elizabeth A. Beverly, Jane Hamel-Lambert, Laura L. Jensen, Sue Meeks, Anne Rubin

**Affiliations:** 1 Department of Family Medicine, Ohio University Heritage College of Osteopathic Medicine, Athens, OH 45701 USA; 2 The Diabetes Institute, Ohio University, Athens, OH 45701 USA; 3 Department of Pediatric Psychology, Nationwide Children’s Hospital, Westerville, OH 43081 USA; 4 Department of Clinical Pediatrics, Ohio State University, Columbus, OH 43210 USA; 5 Community Service Programs, Ohio University Heritage College of Osteopathic Medicine, Athens, OH USA; 6 Southeastern Ohio Legal Services, Athens, OH USA

**Keywords:** Diabetes, Patient navigation, Qualitative research, Process evaluation, Nursing

## Abstract

**Background:**

Diabetes in the United States has reached epidemic proportions and the people of Appalachia have been disproportionately affected by this disease. Strategies that complement standard diabetes care are critically important to mitigate the risk of complications, reduce health expenditures, and improve the quality of life of patients living in rural Appalachia. The purpose of this study was to conduct a qualitative process evaluation of a patient navigation program for diabetes after its first year of implementation.

**Methods:**

The process evaluation assessed how the Diabetes Navigation Program was delivered as well as how it was experienced by the navigators, providers, health administrators, and office staff at an endocrine specialty center in rural Appalachian Ohio. We employed total population sampling to conduct in-depth, face-to-face interviews with all providers, health administrators, staff, and navigators at a Diabetes Endocrine Center. Interviews were transcribed, coded, and analyzed via content and thematic analyses using NVivo 11 software.

**Results:**

Seventeen individuals (providers *n* = 5, health administrators *n* = 4, office staff members *n* = 3, and navigators n = 5) participated in in-depth, face-to-face interviews (age = 44.7 ± 11.6 years, 82.4% female, 94.1% white, 13.3 ± 9.6 years work experience). Fidelity of implementation: The navigation team carried out most of the activities denoted in the Work Plan, therefore the program was implemented somewhat successfully. Qualitative analysis revealed three themes: 1) The navigator addresses sources of health disparities: All participants described the role of the diabetes navigator as someone who is knowledgeable about diabetes and able to identify and address health disparities. 2) The navigators are the eyes in the community and the patients’ homes: Navigators offered providers and clinic staff a rare glimpse into the personal lives of patients, which led to the identification of unrecognized barriers. 3) Difficulties with cross-system integration of services: Differences in the organizational culture and vision of the specialty center and navigation office contributed to systemic barriers.

**Conclusions:**

Overall, this process evaluation highlights the importance of coordinating providers, health administrators, medical office staff, and navigators to address barriers to diabetes care. Forthcoming research is needed to document the clinical effectiveness and sustainability of the Diabetes Navigation Program in rural Appalachia.

## Background

Diabetes in the United States (US) has reached epidemic proportions and the people of Appalachia have been disproportionately affected by this disease. Appalachia is a 205,000-square-mile region that encompasses 420 counties in 13 states from New York to Mississippi and includes 32 counties of Ohio [1]. The Appalachian Region is 42% rural, compared to 20% of the US as a whole, and predominantly white (94.6%) [1]. The people in this region battle a poverty rate 1.5 times that of the US average, and suffer from higher unemployment, lower educational achievement, generally poorer health, and lower access to health care [1–3]. In rural southeastern Ohio, diabetes rates far exceed both the national (19.9% vs. 9.4%) [4, 5] and state prevalence (11.0%) [6]. Here, diabetes patients are more likely to have a delayed diagnosis, limited access to health care, lower health literacy, and lower empowerment [7, 8]. Moreover, the Appalachian counties located in southeastern Ohio are designated as economically “distressed,” with nearly a third of residents living below the poverty line [1]. For these reasons, patients are more likely to suffer from macrovascular (i.e., cardiovascular disease) and microvascular complications (i.e., retinopathy, nephropathy, neuropathy), adult-onset blindness, lower limb amputation, food insecurity, and depression [8–11]. Thus, strategies that complement standard diabetes care are critically important to mitigate the risk of complications, reduce health expenditures, and improve the quality of life of patients living in rural Appalachian Ohio.

To address these health disparities in the region, we designed the Diabetes Navigation Program. We selected the Patient Navigation model based on empirical evidence demonstrating reduced barriers and improved outcomes for cancer care in marginalized populations [12–14]. Internationally, navigation programs have been developed to address barriers to timely and effective care in underserved populations [15–27]. Patient navigators are trained personnel, with or without a healthcare background, who engage patients on an individual basis to determine barriers to accessing care or following treatment recommendations, and provide information and services relevant to overcoming modifiable barriers, improving access to care, and facilitating self-management [28]. Patient navigators can be nurses, social workers, community health workers, and peers. Navigation addresses targeted barriers via the provision of services that may include assistance with insurance coverage [29], addressing financial barriers [30], removal of medical system barriers [30], disease-specific education [31, 32], health system education [31, 33, 34], care coordination [31], referral to community resources [32], and emotional support. Whether utilizing nurse navigators, social workers, community health workers, or peers, the evidence suggests that navigator services have broad implications for a variety of healthcare issues, including early screening and treatment for chronic disease, improved clinical outcomes, increased clinic attendance, and reduced hospital admissions and readmissions [15–27]. For the Diabetes Navigation Program, we employed nurse navigators given the importance of understanding the complexities of diabetes and its management.

Diabetes navigation programs have shown reductions in A1C levels [35–42], reduced hypoglycemia [43], increased medical visits to providers [23, 43], reduced hospitalization/emergency department utilization [37, 43, 44], increased diabetes knowledge [36, 38], and increased diabetes self-efficacy [35, 38, 42]. Further, diabetes navigation programs are feasible with high rates of satisfaction and relatively low costs per person [45, 46]. However, to our knowledge, no patient navigation programs have employed registered nurses to address the complexities of diabetes management. Therefore, our Diabetes Navigation Program is the first of its kind to coordinate registered nurse navigators and providers to navigate diabetes patients through and around barriers in the healthcare system to reduce health disparities and ensure timely treatment. Thus, if successful, the implementation of Diabetes Navigation Program may be a promising model to address sources of health disparities in a rural, underserved setting.

As a first step in assessing the feasibility and effectiveness of the Diabetes Navigation Program, we conducted a qualitative process evaluation to assess how the program was delivered as well as how it was experienced by the navigators, providers, health administrators, and office staff of an endocrine specialty center in rural Appalachian Ohio. Process evaluations allow researchers and providers to gain insight into the best practices of the program in order to ensure its effectiveness and sustainability over time [47]. Thus, to support the advancement of the Diabetes Navigation Program, we conducted this evaluation to learn about its successes and challenges after its first year of implementation.

## Method

### Context – A detailed description of the diabetes navigation program

The Diabetes Navigation Program was a feasibility study designed to produce a set of findings to help determine whether an intervention should be recommended for efficacy testing of nurse navigation for diabetes [46]. The goal of the Diabetes Navigation Program was to improve health outcomes and lower health care expenditures for individuals with diabetes. We aimed to achieve these goals by expanding access to care and enhancing care coordination via nurse navigators. Our Diabetes Navigation Program shared the principles of Harold P. Freeman’s model of patient navigation, with an intent to promote timely movement of patients through the fragmented healthcare system and eliminate barriers to diabetes care. The nurse navigators addressed the financial, communication, structural, emotional, and sociocultural barriers that prevent or delay timely care. For example, navigators explained diagnostic reports to patients, served as a consistent point of connection, accessed insurance benefits by filling out paperwork and making phone calls, referred patients to legal services at a civil legal aid firm, referred patients to mental health providers and specialty providers, increased food stamps by filling out paperwork, delivered emergency food boxes, found permanent or temporary housing by contacting Housing and Urban Development agencies, contacted Home Energy Assistance Program programs, provided diabetes education, reduced hospital bills through Hospital Care Assurance Programs, distributed diabetes medication at no or reduced cost, obtained transportation services through public insurance programs, attended medical visits, and offered emotional support. By removing barriers to everyday living, such as having electricity for a refrigerator to store insulin, navigation helps patients with diabetes focus on their diabetes and participate in healthcare decisions. The nurse navigators did not provide any clinical care to patients, rather strictly navigation services.

The target population for the Diabetes Navigation Program included individuals with type 1, type 2, and gestational diabetes receiving diabetes care from the Diabetes Endocrine Center. The Diabetes Navigation Program serves patients in Athens, Hocking, Meigs, Morgan, Perry, and Washington counties in Ohio. Diabetes rates are significantly higher in each of these counties) [5], compared to the United States prevalence [48]. Recent county health rankings show that the southeastern Appalachian region of Ohio ranks in the bottom half of poorest health outcomes [49]. Further, these counties are designated as health professional shortage areas (HPSA), with no diabetes specialists or hospitals in Perry or Morgan counties [50]. Approximately 46,000 diabetes patients live in these six counties.

The Diabetes Endocrine Center is recognized as the region’s major diabetes management and patient care facility as well as an ever-expanding comprehensive clinical research facility for obesity, diabetes, and other metabolic diseases. Care at the center is team-based and patient-centered. The physicians work in concert with nurse practitioners, pharmacists, clinical psychologists, certified diabetes educators, dietitians, and clinical research nurses. All of these units are housed in the same facility for the ease of access for the patient. This provides an integrated interprofessional clinical experience and a rich learning environment for future health care professionals. During Year 1 of the Diabetes Navigation Program, the clinic treated 2124 patients for a total of 5866 visits. Providers at the Diabetes Endocrine Center referred patients with A1C levels > 7.0% with one or more health disparities to the Diabetes Navigators.

### Program leadership

The Principal Investigator (EAB) of the Diabetes Navigation Program was responsible for oversight of all programmatic activity and fiscal responsibilities, including contracting, budget monitoring, implementation of proposed activities to accomplish stated objectives and goals, completing strategic planning, and conducting the systematic evaluation of the program to assess the process and outcomes. A nurse manager (SM), who has been doing family navigation for over 20 years in the region, hired and trained all of the navigators. A job description for a full-time equivalent (FTE 1.0) nurse navigator supported by external grant funding was written and posted online via the University website. Registered nurses from Appalachian Ohio were given priority. Four other nurse navigators were currently employed by the University in the Family Navigation Program in the Community Clinic at the University’s medical school building. These navigators included a navigator nurse manager (SM), two nurse navigators (FTE 1.0) specializing in high risk pregnancies, and a part-time diabetes navigator (FTE 0.5) specializing in children and type 1 diabetes. The external grant funded the full-time diabetes nurse navigator (FTE 1.0) and 0.1 FTE of the navigation nurse manager. The nurse manager then supervised the navigators to ensure that the principals and standards of services were consistent with the Patient Navigation Model [13]. Training of the newly hired diabetes nurse navigator followed an apprentice-based model, which included shadowing the three navigators and working closely with the navigation nurse manager. In addition, the five navigators worked in close proximity at the University in an office suite and could problem-solve challenges collectively. No formal curriculum or training was utilized to train the nurse navigator.

### Program recruitment

The navigation leadership team (EAB, JHL, SM, AR) met with local diabetes providers to tell them about the Diabetes Navigation Program and the services that were provided at no cost to the patient. They encouraged the five providers (three physicians, one nurse practitioner, one certified diabetes educator) from the Diabetes Endocrine Center to refer patients who were struggling with glycemic control and diabetes self-management. In addition, they encouraged providers to refer patients with barriers, such as housing issues, transportation, food insecurity, no or lack of insurance coverage, to the program. Providers were instructed to make the referral based on whether or not he/she felt that the patient was reaching individualized diabetes targets as well as to address specific health disparities. Providers asked the patient for permission to provide the referral and the patient signed a release of protected health information form. All pertinent medical information (e.g., A1C, blood pressure, lipid profiles, body mass index, diabetes complications) was sent to the Diabetes Navigation Program with the referral. Initially, providers were instructed to send all referrals from via facsimile (fax) machine. We planned to gain access during Year 1 to the Electronic Health Record (EHR) system from the Diabetes Endocrine Center to improve workflow and communication. If a patient contacted the Diabetes Navigation Program as a self-referral, we obtained permission from the primary care provider for the referral and had the patient sign the release of protected health information form so that we could obtain the necessary medical information.

All five providers from the Diabetes Endocrine Center referred patients to the Diabetes Navigation Program. From October 2015 to October 2016, a total of 39 diabetes patients (mean age = 58.5 ± 16.9, diabetes duratio*n* = 12.5 ± 9.0, A1C = 8.9 ± 2.3% or 11.6 ± 1.1 mmol/l, BMI + 36.5 ± 9.1) received navigation services from the Diabetes Navigation Program during its first year of implementation. The patients’ most common barriers to diabetes care included finances (84.6%, *n* = 33), food insecurity (76.9%, *n* = 30), mental health issues (71.8%, *n* = 28), vision problems (53.8%, *n* = 21), transportation (41.0%, *n* = 16), lack of social support (38.5%, *n* = 15), housing (30.8%, n = 12), legal issues (30.8%, n = 12), literacy (15.4%, *n* = 6), and domestic violence (10.8%, *n* = 4). Length of involvement ranged from 1 day to 12 months. No minimum or maximum number of touch points (in-person meetings or phone calls – we did not document email or text message exchanges as visits) with the patients was set for the program; all interactions were based on patient need. The range in visits for Year 1 was 2 to 73 (mean = 14.7 ± 10.8). Navigators met with the patients at their homes, public spaces (e.g., libraries, restaurants), physician offices, and the navigators’ offices. At the initial visit, the navigator conducted an intake lasting approximately 2 h (e.g., similar to a complete health history in nursing), which included an authorization of protected health information, a personal history, list of family and social contacts, identification of current barriers to diabetes care, diabetes health history, past medical history, medication list, depression screening (Patient Health Questionnaire 9 [51]), diabetes distress (Problem Areas in Diabetes 5 [52]), diabetes self-care (Self-Care Inventory-Revised [53]), and a diabetes care plan checklist (i.e., reinforcing diabetes education, reviewing scheduled medical appointments, addressing diabetes self-care plan, setting individualized diabetes self-care goals). Follow-up visits were scheduled based on individual needs and urgency of issues. Navigation goals were tailored to each individual patient. Services continued until the goals or needs were met; if a goal or need could not be met, the patient was informed why this was not possible (e.g., unable to stop a house foreclosure). Our program did not have a formal termination of services protocol at the start of the program; we planned to develop a protocol over time that it was informed by patient preferences.

### Process evaluation

A qualitative process evaluation was conducted to achieve a deep understanding of the experiences and views of the Diabetes Navigation Program from the perspective of the providers. Our research questions were as follows: 1) Was the Diabetes Navigation Program implemented as designed? 2) What was the role of the diabetes navigator? 3) What were the early successes of the program? 4) What were the ongoing challenges of the program? From October 2016 to December 2016, we interviewed providers, health administrators, and office staff members from the Diabetes Endocrine Center in addition to the diabetes navigators about the successes and challenges of the Diabetes Navigation Program in its first year of implementation. Inclusion criteria for the process evaluation included direct or indirect contact with the Diabetes Navigation Program. We recruited participants via email. The Ohio University Institutional Review Board approved the study (reference IRB protocol number 16 N23). All participants provided written informed consent prior to participation and received compensation for their time.

### Sample

The required sample size in qualitative research relies on the quality of the of the information obtained per sampling rather than the number [54]. The logic of qualitative sampling rests not on generalizability or representativeness, but on the notion of saturation, that is, the point at which no new information is obtained. Therefore, sample size is not a criterion for evaluating the rigor of the sampling strategy but, rather, for evaluating the adequacy and the comprehensiveness of the findings [55]. We employed total population sampling, a type of purposive sampling, where the entire population is included in the sample because they have a particular set of characteristics (e.g., specific experience with the Diabetes Navigation Program) [55]. Therefore, we interviewed all of the providers at the Diabetes Endocrine Center who made referrals to the navigators, the office staff who assisted with the referral process and medical chart documentation, the administrators who oversaw the practice, and the navigators. We made the decisions to interview all five navigators, including the three navigators not funded by the external grant, because they assisted with patients who were pregnant (*n* = 2) and patients who had type 1 diabetes (*n* = 4) during Year 1 of the program.

### Data collection

The multidisciplinary research team devised and field-tested a semi-structured interview guide with two individuals (see Table 1). An experienced qualitative researcher (EAB) conducted all interviews, asking participants broad, open-ended questions about the role of the diabetes navigator, experiences with the diabetes navigation, barriers to implementation, and early successes with the program. The interviewer used directive probes to elicit additional information and clarify questions. Interviews were conducted at the Diabetes Endocrine Center and University conference rooms, and lasted 20–90 min. All interviews were digitally audio-recorded and transcribed verbatim. The researchers performed quality checks of the transcribed files while listening to the interview recordings to validate the transcriptions. Participants’ names and identifiers were removed to protect patient confidentiality.

**Table 1:** Interview Guide Questions

1. In your own words, what is diabetes navigation?	
2. What qualities make a good diabetes navigator?	
3. How might diabetes navigation help patients struggling with their diabetes management?	
Probe: Please provide examples of diabetes navigation successes.	
4. Please describe your experience with diabetes navigation at the Diabetes Endocrine Center?	
Probe: How does diabetes navigation help providers in the Diabetes Endocrine Center?	
Probe: How does diabetes navigation not help providers in the Diabetes Endocrine Center?	
6. What barriers have you experienced with diabetes navigation?	
7. What is needed to improve the diabetes navigation program at the Diabetes Endocrine Center?	
Probe: What is the diabetes navigation program doing well?	
Probe: What is the diabetes navigation program not doing well?	
Probe: How do you propose we improve the diabetes navigation program?	
8. Do you have any other comments or suggestions about the diabetes navigation program?	

Additionally, a reflexive journal (written by the interviewer) was maintained during data collection. The purpose of the reflexive journal was to record the interviewer’s thoughts, beliefs, and experiences during the research process. The personal notes were used to evaluate the investigator’s response to specific interviews. The value of the reflexive journal was to reduce personal bias and maintain objectivity [56]. In addition, memoing was incorporated to record ideas and insights regarding the data. Memoing is a form of data coding completed during data collection. Memos were written to connect notations with shared meaning. Such reflections helped the interviewer recognize the need for additional interviewing of the participants [57]. Salient information from the reflexive journal and memoing was integrated into the data analysis.

### Data analysis

The multidisciplinary research team consisted of a diabetes behavioral specialist and qualitative methodologist, a clinical psychologist, a public health professional, a registered nurse, and a medical legal provider. Two researchers (EAB, LLJ) analyzed the data using standard qualitative techniques [58]. Specifically, the researchers performed content analysis by independently marking and categorizing key words, phrases, and texts to identify codes to describe the overarching themes [59]. Transcripts were coded and then reviewed to resolve discrepancies. This process continued until saturation was reached; that is, until no new codes emerged. After all transcripts were coded and reviewed, one member of the research team (LLJ) entered the coded transcripts in NVivo 11 software (QSR International, Victoria, Australia) to organize the data to support thematic analysis.

### Rigor

To support credibility (validity), we conducted member checks with five participants to confirm participant corroboration [60, 61]. To support transferability (external validity), we described in detail specifics of the Diabetes Navigation Program, our research questions, and the evaluation methodology so that the findings are comparable to other programs [61]. To support dependability (reliability) of the data, an external researcher not involved in the data collection or analysis performed a data audit by reviewing the findings to achieve researcher corroboration [61]. Finally, to support confirmability (objectivity), we tracked the decision-making process using an audit trail [62, 63]. The audit trail is a detailed description of the research steps conducted from the development of the project to the presentation of findings [62, 63].

## Results

Seventeen individuals (providers *n* = 5, health administrators *n* = 4, office staff members *n* = 3, and navigators n = 5) participated in in-depth, face-to-face interviews (age = 44.7 ± 11.6 years, 82.4% female, 94.1% white, 13.3 ± 9.6 years work experience; Table 2). Below we present the fidelity of implementation followed by the themes addressing the role of the navigator, early successes of the program, and ongoing challenges of the program. Transcript identifiers are used with quotations indicating participant number and position.

**Table 2:** Participant Demographic Characteristics (*n* = 17)

	Participants n (%)
Age (years)	44.7 ± 11.6
Gender	
Female	14 (82.4)
Male	3 (17.6)
Race	
White/Caucasian	16 (94.1)
Mixed	1 (5.9)
Position	
Navigator	5 (29.4)
Provider	5 (29.4)
Administrator	4 (23.5)
Office Staff	3 (17.6)
Work experience (years)	13.3 ± 9.6

### Design

The Diabetes Navigation Program was a single-arm, repeated-measures pilot and feasibility study. Prospective participants were identified by providers at the Diabetes Endocrine Center. Providers referred patients who were not reaching glycemic targets or patients who needed help with barriers to self-care. All patients provided written informed consent and signed a release of protected health information. Participants meeting study inclusion criteria completed a baseline assessment protocol. Those enrolled received diabetes navigation services as needed. Participants completed follow-up assessments at 1-month and 6-months. The Diabetes Navigation Program was a 3-year study. A sample size of 150 was the recruitment goal.

### Fidelity of implementation

As denoted in Table 3, the Work Plan for the Diabetes Navigation Program included activities to be accomplished in Year 1 along with anticipated dates, outcomes/results, evaluation/measurement, and partner responsible. The team successfully established the intake assessment, authorization for protected health information, and referral procedures as well as the Institutional Review Board application and consent process. Unfortunately, due to an impending merger between two health care systems, the navigators were not granted access to the EHR system and we had to continue with the fax referral system; medical chart information was sent to navigators via fax; thus this outcome was not achieved. A total of 49 patients were referred to the navigator in Year 1. Ten patients refused services so exactly 80% of the patients referred were successfully engaged in navigation services; thereby hitting our targeted outcome in the Work Plan for Year 1.

**Table 3:** Summary of Work Plan for Year 1 of the Diabetes Navigation Program

GOAL: We will establish a Comprehensive Diabetes Patient Navigation Program for Rural Appalachians to improve health outcomes and lower health care expenditures for individuals with diabetes through the development and coordinated implementation of the Diabetes Patient Navigator Program to impact the health care delivery system, individual patients, and inform policy.				
Objective One: Establish a Diabetes Patient Navigator Program that serves individuals with diabetes to improve health measures in diabetes clients by addressing barriers to health care and self-care activities Evaluate Annually: # provider/staff trained to identify and refer patients with diabetes due to poor glycemic control; number of referrals received, % of referred individual who engage navigation services; number and type of barriers identified; number of barriers targeted for interventions; number of barriers resolved; repeated measures of patient health metrics including haemaglobin, blood pressure control (all available), depression symptoms, distress symptoms, self-care (intake, 6 m, 12 m); provider and patient satisfaction (annual). Patient admissions, readmissions and emergency department utilization, annual expenditures; patient improvement measures tracked. Healthy People 2020: Improve glycemic control; improve blood pressure control; complete dental, eye, foot exams; increase number performing daily self-monitoring of glucose and getting formal diabetes education.				
Activities Year One: October 2015–October 2016	Dates	Outcome/Results	Evaluation/Measurement	Partner Responsible
Year 1, Activity 1: Design intake, referral procedures, HIPAA compliant releases at Diabetes Endocrine Center (SYSTEM CHANGE)	May 2015–July 2015	• Intake and referral processes in place; staff trained • Navigator to serve Diabetes Endocrine Center • Obtain access EHR at both	• Workflow within health care practice is reformed to screen and refer patients to Diabetes Navigator • Number staff trained • Referrals made	Diabetes Navigators; Medical practice managers
Year 1, Activity 2: Submit protocol to IRB for approval; consent process established (EVALUATION)	August 2015–September 2015	• Consent forms and measurement tools selected • Data collection processes set	• IRB approval received	Principal Investigator
Year 1, Activity 3: Direct services provided to individuals referred to Diabetes Navigator. (INDIVIDUAL CHANGE)	October 2015–October 2016	• 80% of patient referred are successfully engaged in Navigation services • 90% barriers targeted for intervention that the consumer agreed to address with the Navigator are resolved.	• Process: number and types barriers identified and resolved. Goal to see 50 patients. • Health Outcomes: haemoglobin A1C, blood pressure, exams depression, distress, self-efficacy, satisfaction metrics 3 times year • Cost Outcomes: admissions, readmissions, and ED utilization rates tracked; annual expenditures	Diabetes Navigators
Year 1, Activity 4: Manager of Navigator Program facilitates the coordination of all navigation programs (SYSTEM CHANGE)	October 2015–October 2016	• Protocols and policies in place to differentiate types of navigation services and access • Best linkages of care for patients	• System integration increases the capacity and efficiency of service delivery; Single point of referral established	Diabetes Navigators
Years 1,2,3, Activity 5: Diabetes nurse navigators initiate clinical activity to become Certified Diabetes Educator	Jan 2015-April 2018	• Clinical hours accrued	• Certified Diabetes Educator earned at end of Year 3	Diabetes Navigators
Year 1, 2, 3; Activity 6: Diabetes Navigator and manager participate in consortium members meeting to discuss integration efforts, monitor challenges, improve practices; facilitate integration into Diabetes Institute; develop five year strategic plan.	October 2015–April 2018	• Consortium meetings held quarterly • Strategic planning sessions held • Integration of consortium into larger delivery system and Diabetes Institute	• 100% attendance • Steps identified to integrate with Diabetes Institute • Five year strategic plan written such that it situates to strategic initiatives of consortium partners; and adopted by consortium	Diabetes Navigators, Principal Investigator

The diabetes navigator had a caseload of 39 patients at year’s end rather that the anticipated 50 patients (78% of target met). Thus, we did not hit our process outcome. The nurse navigator provided emotional support (59.0%, *n* = 23) to the patients, increased insurance coverage (53.8%, *n* = 21), food stamps and/or emergency food boxes (38.5%, *n* = 15), diabetes supplies (23.1%, *n* = 9), medical referrals (23.1%, n = 9), reduced hospital bills (17.9%, *n* = 7), diabetes education (15.4%, *n* = 6), legal referrals (12.8%, *n* = 5), permanent or temporary housing (10.3%, *n* = 4), and transportation (10.3%, n = 4), utility repairs (7.7%;,*n* = 3). The navigator was able to address 90% of barriers for 35 of the 39 patients (89.7%). The manager of the navigator program coordinated all of the navigators and put in place protocols and policies to differentiate navigation services for the navigators who specialized in high-risk pregnancies as compared to the navigator who specialized in adult diabetes as compared to the navigator who specialized in children with diabetes. Finally, the navigators participated in continuing education unit courses and regular progress meetings with the Diabetes Endocrine Center staff, providers, and administrators. While the program successfully established intake and referral processes and engaged 80% of the referred patients, the program did not hit the patient enrollment target or gain access to the EHR. Further, the navigators were not able to resolve 90% of the barriers for four patients. Therefore, the Diabetes Navigation Program was implemented only somewhat successfully.

### The role of the navigator

#### Theme 1: The navigator addresses sources of health disparities

All participants described the role of the diabetes navigator as someone who is knowledgeable about diabetes and able to identify and address health disparities. Common sources of health disparities discussed by the participants included housing issues, food insecurity, transportation barriers, and financial/insurance barriers. As articulated by one of the navigators:

“Navigation from the stance of a registered nurse is meeting clients where they are in their stage of development and health, and providing them with the tools they need to optimize outcomes. I think navigation involves assessing needs in their social life, in their medical life, mental health, social determinants of health issues, all of those things I think come into play…With our clients, we find that many have no housing or poor housing, substandard housing. Some are in unsafe situations. Some have food insecurity. Some have difficulty with transportation. Some have educational barriers including an inability to read medical text or forms. Some have no insurance, no money to get things that aren’t covered by Medicaid, a lot live in risky environments. Many have substance abuse.” [ID 9, Navigator].

Providers, administrators, and office staff from the Diabetes Endocrine Center stressed the complexity of diabetes management and explained that diabetes navigation filled in “gaps” or “holes” to standard clinical care. As the following administrator and provider shared:

“Diabetes is a pretty complex condition. There’s obviously certain clinical, individualized types of needs somebody has with diabetes, but beyond that diabetes touches so many parts of your lives. To be able to manage diabetes, you have to sometimes have assistance beyond some of the clinical – standard clinical types of care. So, to me, diabetes navigation is filling in all the other holes left to manage diabetes beyond clinical care.” [ID 6, Health Administrator]

“I see Diabetes Navigation as helping stand in the gap between services as usual that are available through standard medical practice and the other aspects of our life that directly impact our quality of life, and our health, and our ability to make it day-to-day. And I see the Diabetes Navigators as helping folks navigate that intersection between what they need to do for their medical health, but also how that interfaces with their day-to-day lives and the multiple stressors they meet.” [ID 12, Provider]

In addition, they discussed the navigators’ ability to travel to and from patients’ homes to identify and address barriers to diabetes management. These participants recognized that navigators provided services beyond what was conceivable at the Diabetes Endocrine Center, as demonstrated by the following two quotations:

“The navigators go to the patients’ houses to see if they have anything in their house – like if they would need a refrigerator. They can go over there and look at their food, they can go over their food, what kind of living [arrangements], what kind of housing. They can help with a patient that is having trouble reading, trouble with transportation, trouble with food, trouble with electric. If they just need more information on nutrition and they don’t have the transportation to get here, the diabetes navigators can go to their home to help them there, or just meet them someplace.” [ID 3, Office Staff]

“A navigator who is trained in healthcare of some sort goes and identifies needs of diabetes patients, whether it be financial or transportation, food acquisition. Some way to improve their care in a way that we aren’t able to in the clinic.” [ID 17, Provider]

### Early successes

#### Theme 2: The navigators are the eyes in the community and the patients’ homes

All participants reported early successes with the Diabetes Navigation Program, from improved glycemic control to increased follow-up to learning about the patients’ lives. For example, while only 17 of the 39 patients returned for a 6-month follow-up visit, these patients showed a significant improvement in A1C from baseline to 6-month follow-up via Wilcoxon Signed-Rank Test (mean change: − 0.79% or − 1.3 mmol/l, Z = − 2.131, *p* = 0.033). Collectively, participants also acknowledged that navigation provided information about barriers that patients typically did not disclose during medical visits. For example, one navigator described a difficult home situation coupled with challenges administering insulin:

“I can tell you about one patient…she was referred because her blood sugars were out of control…I met her at the clinic she had bruising all over one side of her face and of course the first thing I did was ask her about the bruising. She was being abused by the brother that she was living with at the time. So number one thing was to get her out of that situation, but in doing that I discovered… I took her out to lunch while we were waiting to get her into a shelter and found out that she was not able to add together, the sliding scale together with the other dose. Plus, her eyesight was so poor she could not read what she was drawing up…So we were able to discover things that the clinics can’t or don’t have time to do.” [ID 10, Navigator]

Providers, in particular, valued the insight navigation afforded them regarding their patients’ personal lives, as demonstrated by these two quotations:

“We depend very much on this outreach and many times it’s important to understand the patients’ circumstances so I view them as our eyes in the community and the patient’s home because it helps us understand obstacles that people face and that they don’t necessarily share during the medical interview and the history.” [ID 2, Provider]

“I think the Diabetes Navigators can be really helpful in bringing the rest of that person into the room with the physician, that this person is not just diabetes with this A1C level, but this is a person who is also a caregiver and has these challenges and is going back to school, or doesn’t have sufficient access to healthy food, or needs additional transportation supports to make it to their appointments or what have you. So they can help personify and also overcome some of those barriers.” [ID 12, Provider]

Participants described seeing noticeable improvements in patients’ self-care and glycemic control, which they attributed to navigators addressing these barriers via access to community resources and constant follow-up with patients. The most commonly discussed resources included enhanced insurance coverage, free or discounted medications, reduced medical bills, and increased food stamps. As one provider stated:

“I’ve had a couple of patients that really benefitted because they were able to either get more food stamps or they realized they were eligible for Medicaid or they were able to get some resources they may not have had before, which has helped them in other ways because there are a lot of social pieces to diabetes management. And that has helped me in some ways provide better care for my patients.” [ID 17, Provider]

Further, providers, administrators, and office staff perceived that the frequent follow-up, in addition to the resources, encouraged patients to adhere to their self-care regimen (e.g., medication, blood glucose monitoring, clinic attendance). As expressed by the following provider and office staff member:

“I think sometimes what’s really needed with patients is just knowing that somebody is going to be following up on a frequent basis with them instead of once every three months for appointments. And just having a conversation or looking at their blood sugar logs I think is an amazing way to help people stay on track. And so the navigators have really helped with that. Even going to the homes too and finding out what the family situation is like, what the home environment is like and giving us a little bit of insight into challenges.” [ID 16, Provider]

“I’ve seen patients who’ve A1cs that have dropped because of the reminder or now that they can come to their appointments like they should, or they can now afford their medication that they couldn’t because of a program that maybe was related to them, or somebody just to remind them how important it is and why.” [ID 4, Office Staff]

### Ongoing challenges

#### Theme 3: Difficulties with cross-system integration of services

The Diabetes Endocrine Center and navigators serve the same population; however, the differences in organizational culture and vision contributed to systemic barriers. All participants identified the referral system, lack of access to EHR, patient documentation, and physical location as ongoing challenges to the program. The navigators were employed by the University and not the Diabetes Endocrine Center, and therefore, were not located in the center and did not have access to the EHR. These two logistical barriers contributed to challenges with the referral system and documentation of patient visits. To refer patients to the navigator, providers and staff at the center had to fill out a form and submit medical chart information via fax machine in order to protect sensitive patient data. This process created additional work and frustration for the providers and office staff, as one provider expressed:

“The thing that I find as a barrier for me with referring to navigation is I have to run off progress notes and not only fill out the form, but they want some documentation from the chart because the navigator doesn’t have access to it. And that’s ridiculous! And that is a barrier for me. Because I get busy and I don’t have time to go and run off whatever they need. It needs to be more streamlined and easy to make the referral.” [ID 16, Provider]

The fax referral system also led to noticeable delays in receiving referrals. Navigators often received referrals three to five days after the order was placed in the EHR. Moreover, navigators did not always receive medical chart information or the reason for the referral, which left them wanting more information. Navigators explained that they needed to know why providers were referring patients to them so that they could prepare and plan for patient intakes:

“They can be more specific in identifying their needs I think. It would be really nice because I remember getting a lot of referrals that were just a referral with nothing else written on it. I don’t know if we could make it easier by making our referral more specific so that all they would have to do that…If you don’t have a picture before you go in [navigation visit], it makes it twice as hard and twice as long to get that relationship established… It just makes it much more difficult. So I think that is a major thing. Making things really specific as to why they’re doing this referral.” [ID 10, Navigator]

The other main challenge identified by the providers, administrators, and office staff was infrequent documentation of navigation visits. They wanted more consistent updates detailing the navigators’ progress with each patient, including an assessment of a patient’s barriers and the plan to address each barrier. The following quotations from one provider and one administrator communicated these needs:

“When we started doing this, there was no communication back. I would refer someone and not know if they had even touched base with a navigator or what they were doing with them. And it was frustrating… I want to know what issues are identified and I want to know what our plans are to help with those issues. And if it’s beyond what we can do for someone, then say it’s beyond what we can do for someone. So we can better utilize the program for people we know can be helped.” [ID 17, Provider]

“Making sure there is good communication between the navigator and the clinic so that the patient benefits the most from that in the sense that the entire healthcare team understands and knows all the different aspects of what is going on in their care. So it is important that the physician or the nurse practitioner understands and knows that these certain barriers have been taken care of because that might change how they address their care at their next appointment, whether things they choose to address or to congratulate the patient on and encourage them on. So I think communication, making sure that both ways is open and ongoing and documented appropriately.” [ID 14, Administrator]

Also, providers and navigators differed on the frequency and types of documentation notes. Providers wanted frequent updates detailing the navigators’ progress with each patient, following the SOAP (Subjective, Objective, Assessment, and Plan) note format [64]. However, the navigators collected information that was not typically discussed or observed in a clinic setting, and therefore did not conform to standard patient record documentation. As voiced by one of the navigators:

“I believe the challenges related to communication and availability of the navigator stem from the expectation that we would perform as if we were clinic employees adhering to standardized forms, reporting formats, and agency culture. Because we serve patients from multiple counties, we need the flexibility to communicate in a way that provides information that we feel the provider needs to know and is not usually asked in the clinic setting; information often related to social determinants: living conditions, anything leading to the inability of the patient’s ability to adhere to the physician’s directions. Often the format that providers use and prefer allows only for the gathering of information typically expected and gathered in a clinic setting. It is likely that the autonomy needed to best provide information for and about our clients can be challenging for stringently standardized environments and our non-formatted information was sometimes refused and not always provided to the physician.” [ID 7, Navigator]

Further, patient documentation was faxed to the Diabetes Endocrine Center to be scanned into the EHR. Similar to the referral system, the fax documentation system resulted in delays in receiving and updating documentation of navigation visits. All participants agreed that access to the EHR would improve communication and timeliness of the documentation. The following quotations from one provider and one administrator communicated this need:

“I think the quicker the notes can be provided to the physician’s office to be updated in the EHR I think the better off it is. I think a major gap right now, is you have navigators who do not have access to the EHR, so they have to rely on what’s been faxed and a referral. So I see it as we have two separate models right now working.” [ID 1, Administrator]

“Being able to communicate verbally is really, really helpful. But notes would be great too. And if they did have access to the EHR, we could see their notes because it will be in the chart. But right now you have to look for scanned-in documents on the chart. And it’s less likely that you’re going to bump cross it.” [ID 16, Provider]

Finally, all participants expressed a desire to improve communication, and many suggested co-location as a potential solution to address the difficulties with referrals, EHR, and documentation:

“I think that if they were in the office we could just [say], ‘Hey, do you have a minute to touch base with this person?’ It would be easier…I think we would get better communication that way. Because then they could even come tell me, ‘Hey, this is an issue with this patient I just found out about.’ If the navigator was in the office more, we would be able to talk more in real-time.” [ID 17, Provider]

“I think co-location and integration are important. I think they [navigators] need to be physically housed in the space so you can have those warm handoffs when you have someone who is in a situation…You can bring them in the room with you so that they can hear what is going on, they can be a part of the care team…where they’re meeting with the team regularly and having an opportunity for feedback and information back and forth.” [ID 13, Administrator]

## Discussion

The purpose of this qualitative process evaluation was to assess the fidelity of implementation of the Diabetes Navigation Program in rural Appalachian Ohio as well as explore its early successes and ongoing challenges. The detailed description of the program and adherence to the activities listed in the Work Plan indicate that the Diabetes Navigation Program was implemented as intended. Overall, providers, administrators, staff, and navigators agreed that the navigation program was beneficial and necessary. They understood that the role of the navigator was to provide a variety of services to address patients’ health disparities. Further, they agreed that these services filled in gaps in clinical care that providers, administrators, and staff could not address due to time constraints and logistical barriers. For example, navigators could travel to patients’ homes to see their living conditions and what types of foods they had in their refrigerator. This task was not feasible for the providers. In the cancer literature, patients report that navigators fill in gaps in their psychosocial support [65]. As reported in the Work Plan and the interviews, the navigator’s initial assessment of the patients’ clinical and psychosocial well-being took a considerable amount of time and skill, which is not always conducive to a standard medical visit. However, this comprehensive assessment is well-suited to nurses as they are the medical team members with the most face-to-face time with patients. Early successes of the program focused on the navigators’ ability to communicate information about barriers that patients typically did not disclose during medical visits. Providers described navigators as their “eyes to the community and the patients’ homes” to help them provide more comprehensive diabetes care. All of the providers, administrators, staff, and navigators reported improvements in patients’ glycemic control and diabetes self-care, which they attributed to the navigators’ ability to access new benefits and community resources as well as constant follow-up with patients. This was supported with preliminary data from 17 patients with baseline and 6-month follow-up data. Other diabetes navigation programs have shown improvements in A1C levels [35–42, 66]. Additional long-term follow-up data is needed to confirm these improvements in glycemic control and self-care with navigation services, particularly because of the high rate of attrition at 6-month follow-up observed after Year 1 in the study. High rates of attrition have been observed in other diabetes navigation programs [66], this is likely due to the high number of barriers to care. Currently, we are evaluating the clinical (e.g., glycemic control, diabetes self-care behaviors, diabetes distress, depressive symptoms) and health expenditure (e.g., emergency department visits, hospital admissions and readmissions) outcomes of the 3-year Diabetes Navigation Program. These data will be necessary to determine the value, effectiveness, replicability, and sustainability of the nurse-led Diabetes Navigation Program.

**Fig. 1:**
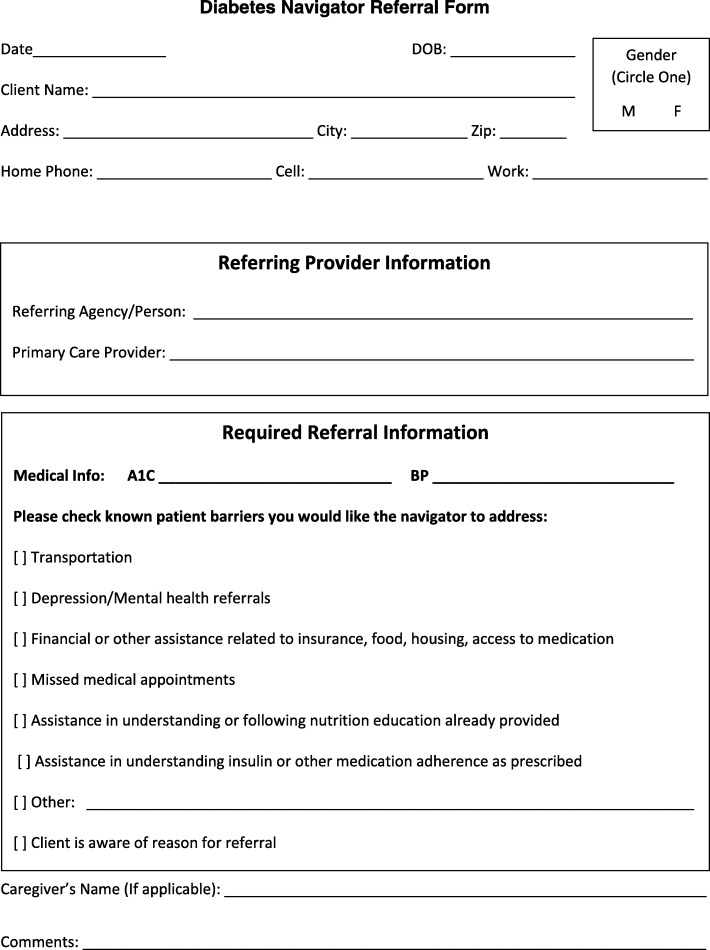
Sample Provider Referral Form to Diabetes Navigator

**Fig. 2:**
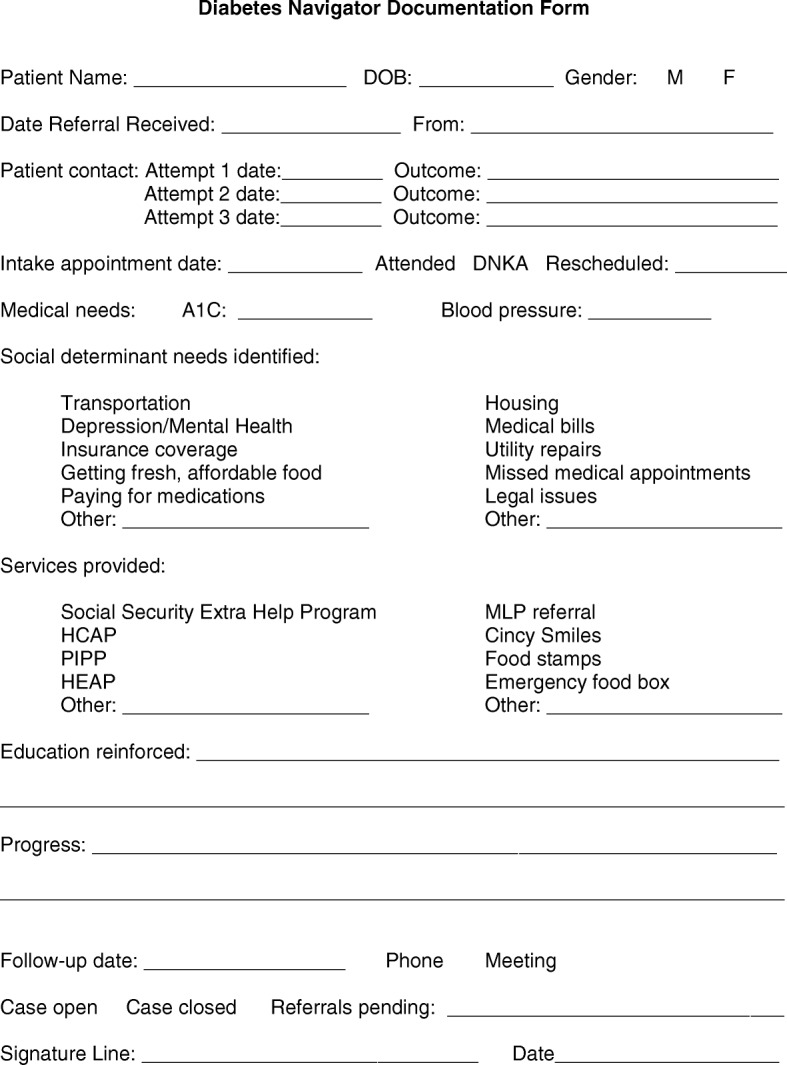
Sample Diabetes Navigator Documentation Form

Ongoing challenges of the program included difficulties with cross-system integration of services. Differences in the organizational culture and vision of the specialty center and navigation office contributed to systemic barriers. Lack of access to the EHR and separate physical locations led to frustration with the referral system and patient documentation. In addition, navigation documentation did not conform to standard patient record documentation (e.g., SOAP note [64]). Thus, an agreed upon system for navigation documentation is necessary to improve efficiency and maintain participant satisfaction. The need for effective and clear referral and documentation processes has been reported in prior navigation research [15, 67–70]. While we were not able to provide access to the EHR or co-locate the navigator in the clinics, we were able to resolve the issues with the referral system and documentation of navigation visits. We revised the referral form to include the reason the provider was referring the patient, health disparities the provider would like the navigator to address, current A1C level, and blood pressure. For the documentation form, we included the patient barriers, the services provided, the diabetes education that was reinforced by the navigator, any progress that was made, the number of visits accompanied with dates, the next scheduled appointment, and the overall status of the case. These forms have been well received by the providers and navigators and we will continue to use them indefinitely (See Figs. 1 and 2). The process evaluation enabled the team to identify challenges to the referral system and documentation of navigation visits, and proactively address them in order to facilitate cross-system integration of services.

### Limitations

Study limitations include the homogeneity of the study sample with regards to setting, sample size, race/ethnicity, and self-reported data. The study was conducted at one endocrine specialty center located in rural Appalachian Ohio, with a small number of providers, administrators, office staff, and navigators. Further, the study sample was predominantly white, which is reflective of the racial and ethnic distribution in rural Appalachian Ohio (94.6% white [1]). Next, self-reported data were vulnerable to social desirability bias. To minimize bias, the researchers informed participants that their responses were confidential and could not be linked back to their personal identity. The researchers also emphasized the voluntary nature of participation and explicitly informed the participants that their responses had no bearing on their employment status. Also, we utilized nurse navigators in our program to address the medical complexities of diabetes and its complications. However, the salary of a registered nurse is substantially higher than a community health worker or peer. Thus, the sustainability of nurse-led navigation program may be difficult. In addition, resource limited areas tend to be health professional shortage areas and staffing a navigation program with nurses may not be feasible. Along those lines, the Diabetes Navigation Program was designed and implemented via a partnership with Ohio University and the Diabetes Endocrine Center in a rural and underserved region in Appalachian Ohio. Most rural and underserved regions in the United States and other countries may not have specialty endocrine centers or universities to help coordinate a navigation program. These regions and countries would benefit greatly from partnerships with local county health departments or ministries. In addition, funding from rural health or global health programs would support additional personnel to provide navigation services and monitor outcomes. Finally, the Diabetes Navigation Program did not utilize a written curriculum to train the nurse navigators or include a formal assessment to assess the navigator’s competency or skill set. Future programs should include written curriculum and competency-based assessments to ensure the integrity of the program.

## Conclusions

Poverty, rural isolation, lack of public transportation, limited specialty providers, fragmentation of care, and a general lack of access to services continue to separate Appalachian families from the services they need. These findings highlight the importance of coordinating providers, health administrators, medical office staff, and navigators to address barriers to diabetes care in rural Appalachia. The qualitative nature of this process evaluation study allowed for an in-depth understanding of the successes and challenges of the Diabetes Navigation Program. These findings may be useful to other providers and researchers involved in designing and implementing patient navigation programs. For example, this study showed that navigators are able to report unrecognized barriers by traveling to and from patients’ homes, access additional benefits and community resources, and serve as a consistent point of connection in between clinic appointments. This study also identified important challenges for providers and researchers to consider; specifically, a mutually beneficial referral and documentation system to facilitate open lines of communication. Finally, long-term follow-up assessing the clinical and health expenditure outcomes is needed to determine the feasibility, cost-effectiveness, and reproducibility of the Diabetes Navigation Program.
